# Isolated Tricuspid Regurgitation: When Is Surgery Appropriate? A State-of-the-Art Narrative Review

**DOI:** 10.3390/jcm14145063

**Published:** 2025-07-17

**Authors:** Raffaele Barbato, Francesco Loreni, Chiara Ferrisi, Ciro Mastroianni, Riccardo D’Ascoli, Antonio Nenna, Marcello Bergonzini, Mohamad Jawabra, Alessandro Strumia, Massimiliano Carassiti, Felice Agrò, Massimo Chello, Mario Lusini

**Affiliations:** 1 Unit of Cardiac Surgery, Fondazione Policlinico Universitario Campus Bio-Medico, 00128 Rome, Italy; r.barbato@policlinicocampus.it (R.B.); chiara.ferrisi@unicampus.it (C.F.); c.mastroianni@policlinicocampus.it (C.M.); r.dascoli@policlinicocampus.it (R.D.); mohamad.jawabra@unicampus.it (M.J.); m.chello@policlinicocampus.it (M.C.); m.lusini@policlinicocampus.it (M.L.); 2 Unit of Cardiac Surgery, Azienda Ospedaliera Maggiore della Carità, 28100 Novara, Italy; antonio.nenna@maggioreosp.novara.it; 3 Unit of Cardiac Surgery, Hospital Santa Maria Della Misericordia, 06129 Perugia, Italy; marcellobergonzini@yahoo.it; 4 Operative Research Unit of Anesthesia and Intensive Care, Campus Bio-Medico University Hospital, 00128 Rome, Italy; a.strumia@policlinicocampus.it (A.S.); m.carassiti@policlinicocampus.it (M.C.); f.agro@policlinicocapus.it (F.A.)

**Keywords:** tricuspid valve, regurgitation, score, valve surgery, valve repair, valve replacement, transcatheter, grading

## Abstract

The increasing interest in tricuspid regurgitation (TR) is due to the deep link between mortality and the severity of TR, as well as the limited application of surgical solutions in a setting marked by high in-hospital mortality, attributed to the late presentation of the disease. This delay in intervention is likely associated with a limited understanding of valvular and ventricular anatomy as well as the pathophysiology of the disease, leading to an underestimation of TR severity. With the rapid development of transcatheter solutions showing early safety and efficacy, there is a growing necessity to accurately understand and diagnose the valvular disease process to determine suitable management strategies. This review will outline the normal and pathological anatomy of the tricuspid valve, classify the anatomical substrates of TR, and present new risk stratification methods to determine the appropriate timing for both medical and surgical treatment.

## 1. Introduction

Right-sided heart diseases have often been overlooked compared to aortic and mitral valve pathologies. Recently, the literature has focused on tricuspid valve (TV) diseases, highlighting positive outcomes for patients undergoing early interventions, either through surgery or transcatheter procedures. The correct timing of intervention is mandatory to improve the patient’s quality of life, reduce symptoms, and decrease mortality rates. Untreated tricuspid valve pathologies, such as severe tricuspid regurgitation (TR), could lead to right ventricular (RV) dysfunction, arrythmias, end-stage heart disease, and death. Nevertheless, the optimal timing for intervention in severe primary TR remains unclear and extremely debated as previous evidence has been gradually replaced over time by less invasive procedures and improved perioperative care, leading to reduced periprocedural risk. In this review, we will focus on current evidence on surgical risk stratification, surgical timing, and the different procedures available for the treatment of TR, trying to shed light on future perspectives for the management of this complex disease [1].

## 2. Anatomy of Tricuspid Valve

The tricuspid valve is a complex anatomical structure that includes leaflets, the tricuspid annulus, chordae tendineae, and papillary muscles. The tricuspid annulus is a saddle-shaped ellipsoid surrounded by anatomical structures, such as the atrioventricular node, the right coronary artery, the coronary sinus ostium, and the non-coronary sinus of Valsalva [2]. When the tricuspid annulus dilates, the anatomic modification of the leaflets primarily occurs along the insertions of the anterior and posterior leaflets, next to the free wall of the right ventricle, as the septal leaflet is relatively fixed at the fibrous trigone [3]. This dilation process leads to the morphological distortion of the annulus in which it becomes more planar and circular [4].

### Morphology of Tricuspid Valve

**Figure 1:**
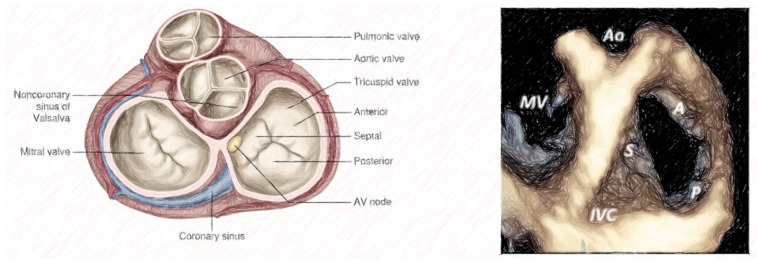
Anatomy of tricuspid valve.

**Figure 2:**
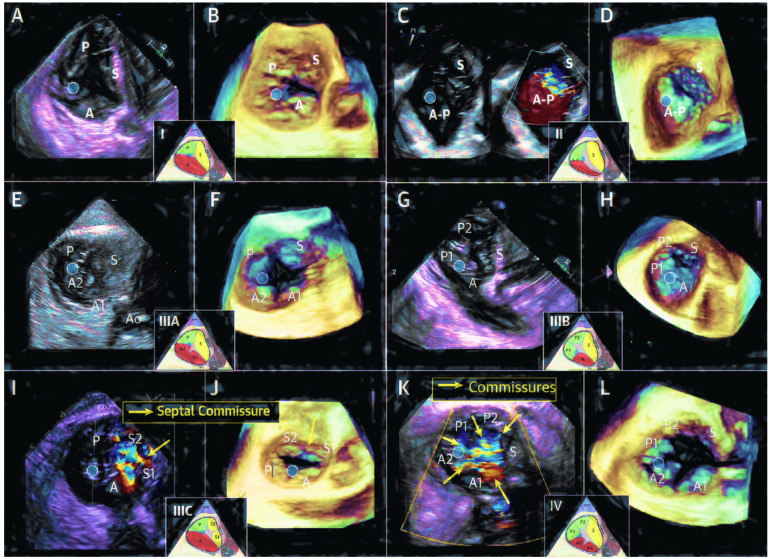
(**A**,**B**) Type I, 3-leaflet configuration; (**C**,**D**) type II, 2-leaflet configurations; (**E**,**F**) type IIIA, quadricuspid valve with 2 anterior leaflets; (**G**,**H**) type IIIB, quadricuspid valve with 2 posterior leaflets; (**I**,**J**) IIIC, quadricuspid valve with 2 septal leaflets; (**K**,**L**) type IV, 5-leaflet configuration. A indicates ¼ anterior leaflet, P indicates ¼ posterior leaflet, and S indicates ¼ septal leaflet [5].

The three leaflets, known as the septal, antero-superior, and inferior leaflets, in anatomical terms, can be identified by three anatomical structures, including (i) the anteroseptal commissure (near the aortic valve); (ii) the interventricular septum; and (iii) the anterior papillary muscle, which is more often fused with the moderator band. Each leaflet is defined based on its attachment points: the septal leaflet is attached to the interventricular septum, the anterior leaflet extends from the anteroseptal commissure to the anterior papillary muscle, and the posterior leaflet extends from the anterior papillary muscle, along the inferior wall of the right ventricle, to the postero-septal commissure. Distinguishing leaflets through echocardiography may be challenging, but it is generally accepted that specific anatomical differentiation may not be necessary for the planning, execution, and outcomes of the procedure. The presence of multiple cords or arches with complex chordal insertions within the leaflets, including commissures and indentations or clefts, can complicate device placement. A separate leaflet is characterized by (i) an independent movement from the adjacent leaflet and (ii) a systolic color Doppler flow, extending into the region around the leaflet. Anterior leaflets are numbered, starting from A1 at the anteroseptal commissure, with the next leaflet being A2 in an inferior/lateral position. The posterior leaflets, instead, are numbered beginning from P1 at the anterior papillary muscle, with the next leaflet being P2. The septal leaflets are numbered starting from S1 at the anteroseptal commissure, with the next posterior leaflet being S2. A recent study analyzed a total of 579 patients from four medical centers (two in Europe and two in the USA) who underwent transesophageal echocardiography (TEE) to assess TV function and morphology. Four different classes were identified according to the leaflet number: (i) type I has three leaflets; (ii) type II has two leaflets; (iii) type III has four leaflets (type IIIA has two anterior leaflets, type IIIB has two posterior leaflets, and type IIIC has two septal leaflets); (iv) and type IV has more than four leaflets. Data show that approximately 39% of patients have four functional valve leaflets, and type IIIB is the most common. The term “mixed disease” is used when a combination of both degenerative disease (such as prolapse or flail) and functional disorder (such as a degenerated leaflet or significant annular dilation) occurred [5] (Table 1; Figure 1 and Figure 2).

## 3. Tricuspid Valve Regurgitation

Since the early 1950s, tricuspid valve regurgitation has been classified into two categories: (i) organic and (ii) functional TR. According to this classification, organic TR occurs when anomalies primarily involve the valve anatomy without left heart disease or pulmonary hypertension, and it represents 8–10% of all severe TR cases [6,7]. It can be further categorized as congenital or acquired TR. The latter includes rheumatic diseases, carcinoid syndromes, infectious endocarditis (IE), and leaflet damages due to implanted devices or repeated endomyocardial biopsy, radiotherapy, and thoracic trauma [8,9]. Severe TR in patients with congenital heart disease may occur as post-procedural damage from corrective cardiac surgery. Functional or secondary TR (FTR) is the most common presentation (>85%) and is associated with annular dilation and/or leaflet tethering [10,11]. Heart diseases and/or pulmonary hypertension are the most common causes. Several studies showed that an RV systolic pressure higher than 55 mmHg may lead to secondary TR, even in the absence of tricuspid valve pathology, whereas significant TR associated with an RV systolic pressure lower than 40 mmHg is more likely to represent a primary valve condition [12].

### 3.1. Classification of Tricuspid Regurgitation

Tricuspid regurgitation is generally classified in primary disease (including leaflet pathology) and secondary disease (including non-leaflet pathology). Secondary TR has traditionally been considered as a distinct entity from primary TR. It is mainly characterized by intact TV leaflets and results from right ventricular remodeling due to pressure or volume overload [13,14] A functional classification, based on leaflet mobility, is used for secondary TR, according to Carpentier. This classification may be applied to primary TR and TR induced by cardiac implantable electronic devices (CIEDs), where different etiologies lead to altered leaflet mobility. This phenomenon was common in traditional CIED implants. The catheters traditionally used could interfere with the normal coaptation of tricuspid leaflets or produce ventricular remodeling. With improvements in technology, leadless CIEDs have been developed with excellent results. Thanks to their ease of implantation and the absence of intracavitary catheters, which eliminates the risk of functional TR, these devices are gradually replacing traditional CIEDs. However, in an overall view of secondary TR, more aspects should be analyzed, such as the coaptation mode, the characteristic changes in the tricuspid annulus (TA), the right ventricle (RV), and the right atrium (RA), suggesting that, based on pathophysiology, “not all secondary TRs are alike” [15,16]. Patients with secondary TR due to pulmonary hypertension or right ventricular myocardial disease often present ellipsoidal right ventricular dilation associated with leaflet tethering and a low grade of left atrial dilation. The severity of atrial secondary TR, instead, characterized by minimal RA and TA area volume, may rapidly progress, leading to adverse outcomes and secondary right ventricular dysfunction [17,18]. Although evidence is scarce, focusing on rhythm control strategies may be useful for reducing atrial secondary TR, reversing atrial and TA remodeling. Moreover, the use of annuloplasty devices may be successful for the surgical repair of leaflet tethering [19,20]. It could be challenging to diagnose the primary cause of TR in advanced stages, especially when long-standing ventricular secondary TR has occurred, developing with atrial fibrillation [21]. Even if the stage is severe, tricuspid regurgitation can endure for an extended period without noticeable symptoms, varying from being asymptomatic to collapse to advanced stages of heart failure. A new clinical classification system has been introduced, dividing TR into five stages according to hemodynamic changes and the patient’s clinical status. Stages 1–2 are characterized by the compensatory mechanism of the heart, including right ventricular remodeling and increased central venous pressure. Patients might be asymptomatic or might show minimal symptoms, which are easily managed by diuretic therapies. Stages 3 and 4 show increased central venous congestion, leading to a deterioration of the patient’s clinical condition, characterized by edema and dyspnea. In the final stage, both the right atrium and ventricle become severely enlarged, potentially causing organ damage such as hepato-renal syndrome, characterized by volume overload and ascites. Typically, in the later stages of the disease, symptoms such as fatigue, dyspnea, and reduced functional capacity become severe, modifying the daily lives of patients. Right heart catheterization studies show all the hemodynamic changes caused by TR. Initially, the right ventricle compensates for the increased volume by enlarging and assuming a spherical shape. As the disease progresses, the reduced forward output and the volume loss from tricuspid regurgitation lead to a decline in overall cardiac function, a reduced peripheral perfusion, and a limited exercise tolerance [22,23,24] (Table 2 and Table 3).

### 3.2. Grading of Tricuspid Regurgitation

The evaluation of the tricuspid valve structure and the flow jet’s characteristics is involved in the qualitative assessment of TR. The presence of several structural anomalies, such as flail or leaflet discontinuity with a large coaptation gap, could be indicative of severe tricuspid regurgitation (TR). The Doppler parameters considered in the qualitative evaluation of TR are (i) the area and the eccentricity of the color flow jet, (ii) the flow convergence zone, and (iii) the continuous wave Doppler jet density. However, the use of the color Doppler jet presents some significant limitations. The jet flow and the color jet area are primarily regulated by the conservation of momentum (generally defined as flow [Q] × velocity [V]). If Q = effective regurgitation orifice area (EROA) × V and jet momentum (M) = Q × V, then M = EROA × V^2^. Therefore, a significant increase in the velocity of the regurgitant jet will affect the area of the color jet; for the same EROA, a TR jet moving at a velocity of 2.5 m/s could have a color jet area that is one-fourth the size of a mitral regurgitation jet with a velocity of 5.0 m/s. Thus, with the use of this imaging technique, it is possible just to diagnose TR, but further quantitative analysis is needed to exactly quantify a central “other than small” TR jet. The shape of the TR can be very complex given the variability of leaflets and commissures’ numbers, making the qualitative and semi-quantitative assessment of TR very challenging [25,26,27]. Therefore, the use of one single measurement of some parameters (i.e., vena contracta diameter) cannot be enough to characterize the jet. Generally, the apical four-chamber view is used in the measurement of VC. However, using the septo-lateral dimension to compute the VC could underestimate its effective dimension because it is often the smallest measure of an elliptical orifice. To solve this issue, the use of the average VC measurement from both the parasternal inflow view and the apical four-chamber view is suggested by some authors, using a 9 mm cut-off to distinguish between moderate and severe TR. Moreover, the shape of the regurgitant orifice and the echocardiographic window used for the analysis can significantly impact the sensitivity and specificity of VC width measurement [28,29].

Some quantitative methods are used to better evaluate the TR: the primary recommended quantitative method is the Proximal Isovelocity Surface Area (PISA), which is based on the principle of conservation of mass. To determine the PISA shell area, the Doppler color baseline is adjusted in the direction of the regurgitant flow. The EROA is obtained by dividing the PISA flow by the peak TR velocity (VTR). The EROA, when multiplied by the TR velocity–time integral (TRVTI), provides a measure of the regurgitant volume (RegVol). However, relying solely on these measurements does not enable the calculation of the Regurgitant Fraction (RegFr) since the total systolic volume is not assessed; this parameter, which has important prognostic value, can be obtained using the RV 3D systolic volume. The PISA methodology presents several limitations. The first limitation is an issue with the Doppler angle effect. As flow approaches a small orifice, the isovelocity surfaces do not present a hemisphere form, but they exhibit a sea urchin-like shape, with a greater surface area than a hemisphere of the same radius, resulting in a 30–35% underestimation of the EROA [30,31,32]. Secondly, as occurs in functional mitral regurgitation, functional TR can be temporally variable and depends on the moment of the echocardiographic measurement, determining the underestimation or overestimation of the EROA. The integration of PISA radii over the systolic time interval could improve the estimation of TR as occurs for the assessment of the ERO in mitral functional regurgitation. Thirdly, the regurgitant orifice is not placed within a planar surface: in the case of a funnel-shaped (e.g., in the presence of flail or prolapse) or wedge-shaped (e.g., marked leaflet tethering) surface, a correction of the leaflet angle may be necessary. Fourthly, when dealing with a hemisphere, the low blood flows within the right heart could determine an underestimation of the PISA because of a smaller difference between the velocity of PISA aliasing and peak TR [33]. It should be possible to adjust the low flow value by multiplying the flow by Vmax/(Vmax − Va), and thus, the EROA calculation should become 2pr2 (Va)/(Vmax − Va).

The reversal of systolic hepatic venous flow is considered a sign of severe TR according to several guidelines. However, its specificity can be low because it can be affected by several factors, including right ventricular diastolic function, the presence of atrial fibrillation, and rheumatoid arthritis [34]. Furthermore, a systolic flow reversal in the hepatic vein can occur in the case of moderate to severe TR [35,36]; this phenomenon can be particularly evident in patients with moderate TR associated with high right atrial or ventricular pressure due to other causes. Hence, it should only be used as a complementary diagnostic tool in clinically evident disease.

The assessment of right ventricular remodeling, including both dilatation and dysfunction, is crucial in patients with clinically significant TR. Measurements of basal and mid-ventricular septo-lateral dimensions and the length from the apex to the tricuspid annulus are typically obtained from a focused RV view, providing larger dimensions compared to apical four-chamber or modified RV views [37,38]. Assessing RV function includes (i) an evaluation of systolic excursion on the tricuspid annular plane, (ii) Doppler systolic velocity, (iii) the change in the fractional area, iv) and global longitudinal strain or free wall of the right ventricle. These measurements could have prognostic value in patients with TR [39,40].

Three-dimensional transthoracic echocardiography (TTE) could present advantages in the quantification of right atrial and ventricular volumes compared to cardiac magnetic resonance (CMR) [41]. In addition, 3D TTE allows for the evaluation of the tricuspid valve tenting volume and the tricuspid annular area. While the CMR tends to overestimate RV volumes, the other parameters are superimposable in the two methods (TTE and CMR) [42,43].

Indexing the right ventricular contractility to afterload, or RV–pulmonary artery coupling, describes a physiological state where the mechanical workload is effectively transferred to pulmonary circulation. In conditions of increased afterload, such as in severe secondary TR, RV contractility may increase to satisfy the demand. As shown in recent studies, the echocardiographic measurement or RV-PA coupling is independently associated with all causes of mortality in patients with severe secondary TR. In severe TR, a Doppler evaluation of pulmonary artery pressure might be underestimated in the case of poor left or right ventricular function, especially compared to invasive measurements [44].

### 3.3. Clinical Implication

Severe tricuspid regurgitation (TR) is associated with poor prognosis, including progressive right ventricular dysfunction, renal and hepatic failure, chronic right heart failure, and an increasing need for diuretics. However, excellent outcomes can be achieved when intervention is performed early in the course of disease, particularly in patients with a low TRI-SCORE (we will discuss this later). Thus, the management paradigm should shift: the poor outcomes observed with ITVS are not inherently due to the complexity of the procedure itself but rather to delayed intervention and the advanced disease stage in many patients. With the rapid advancement of transcatheter techniques, the TRI-SCORE model is emerging as a valuable tool to identify patients that benefit the most from surgical or transcatheter treatments. It is crucial to highlight that the majority of patients with severe TR are still managed conservatively. Transcatheter therapies, as a less invasive alternative to surgery, encourage earlier intervention and an expansion of the treated patient population provided they prove to be safe and effective. Additionally, transcatheter tricuspid valve replacement could address a major limitation of edge-to-edge repair, namely significant residual TR. Ongoing and future randomized clinical trials (TRILUMINATE Pivotal Trial, TRI-FR, CLASP II TR, and TRISCEND II Pivotal Trial) are expected to provide stronger evidence regarding the optimal timing and type of intervention to improve outcomes in this patient population.

## 4. Indication for Surgery

**Figure 3:**
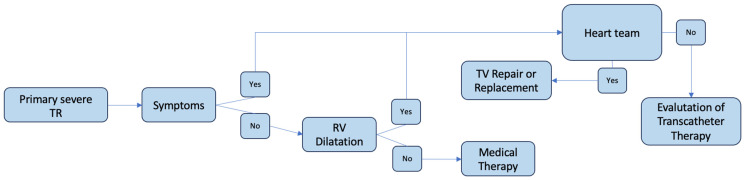
Guidelines for the treatment of TR.

The ESC 2021 guidelines highlight the presence of symptoms and right ventricular enlargement as a priority aspect for treatment. Surgical intervention is recommended for patients “with severe isolated tricuspid regurgitation, who experience severe symptoms or exhibit progressive dilation/dysfunction of the right ventricle” [45,46]. The Guidelines of the American Heart Association/American College of Cardiology (AHA/ACC) agree with the European guidelines. Finally, great relevance is placed on preoperative screening to enhance patients’ outcomes. Recently, European guidelines introduced transcatheter treatment (TTVI) for patients with high surgical risk or those not suitable for surgery. Medication treatments are applied to patients with advanced cardiomyopathy when significant dysfunction of the left or right ventricle or pulmonary hypertension (sPAP > 60 mmHg) occurs [47] (Table 4; Figure 3). The functional anatomy of the tricuspid valve apparatus could be evaluated using three-dimensional transthoracic echocardiography (3D-TTE) and/or three-dimensional transesophageal echocardiography (3D-TEE). The severity of tricuspid regurgitation (TR) can be assessed using semiquantitative color and spectral Doppler parameters. Additional advanced imaging may be useful when echocardiography is inconclusive. Specific signs of severe TR include (i) significant systolic leaflet separation, (ii) systolic hepatic venous flow reversal calculated with pulsed-wave Doppler, and (iii) a triangular continuous-wave TR Doppler signal (with early peaking). Right ventricular and atrial dilation are additional signs. The TR color jet does not measure the regurgitant volume but reflects the jet momentum. Therefore, while a small TR color jet indicates trivial or mild TR and a very large jet is specific to severe TR, patients with pulmonary hypertension (PHT) may present larger jets, overestimating the TR orifice area. Additionally, in severe TR, rapid equalization of right atrial and right ventricular pressures may be associated with non-aliasing jets. It is essential to define quantitative measures of TR severity, including the estimation of the anatomical regurgitant orifice area, by measuring the vena contracta and quantifying the effective regurgitant orifice area (EROA) and regurgitant volume (RVol). Generally, a vena contracta ≥ 7 mm indicates severe TR [48,49], although some studies suggest a threshold of 9 mm based on two orthogonal 2D views. The use of a 3D color assessment to measure the vena contracta width might be more appropriate than a single 2D image because the TR coaptation zone is often non-circular.

A lot of evidence shows that the EROA is particularly linked to adverse outcomes in various settings [50]. The current American Society of Echocardiography (ASE) and the European Association of Cardiovascular Imaging (EACVI) guidelines define severe TR as an EROA ≥ 0.40 cm^2^ and an RVol ≥ 45 mL [51,52,53]. Nevertheless, patients undergoing transcatheter tricuspid valve interventions (TTVIs) often have an anatomical regurgitant area many times greater than an EROA of 0.40 cm^2^; thus, extended classification to include “massive” and “torrential” TR (both associated with adverse outcomes) has recently been proposed [54]. In patients with severe tricuspid regurgitation (TR), a comprehensive assessment of the right ventricle (RV) should be performed in a euvolemic state. This assessment should include standard echocardiographic measurements of RV size and function, as well as the quantification of morphological, functional, and tissue remodeling of the RV [55]. The evaluation of RV strain using 2D echocardiography or cardiac magnetic resonance (CMR) is less influenced by load compared to tricuspid annular plane systolic excursion (TAPSE) in severe TR and is more sensitive in detecting early RV dysfunction and predicting overall clinical outcomes. The RV ejection fraction can be measured using various imaging modalities (CMR, 3D echocardiography, and cardiac computed tomography [CCT]), but it does not account for the relationship between RV contractility and afterload, potentially overestimating RV systolic function in severe TR. The TAPSE/systolic pulmonary artery pressure (SPAP) ratio, a non-invasive marker of RV–pulmonary artery coupling, could overcome this limitation, being a prognostic value in various conditions, including severe TR, in which a TAPSE/SPAP ratio <0.31 mm/mmHg indicates poor prognosis [56,57]. In a recent propensity-matched analysis, patients with moderate RV dysfunction (TAPSE 13–17 mm) seemed to gain better outcomes from transcatheter tricuspid valve interventions (TTVI) [58]. The presence of contractile reserve in response to pharmacological or physical stress is prognostically relevant in patients with pulmonary hypertension and severe baseline RV dysfunction. Hence, further studies are needed to underline the role of stress imaging in severe TR. Right heart catheterization is the gold standard for assessing the severity and mechanism of pulmonary hypertension (PHT), pulmonary vascular resistance, the right atrial (RA) pressure/pulmonary capillary wedge pressure ratio, the pulmonary artery pulsatility index, and PHT reversibility [58]. Additionally, a TAPSE adjusted for afterload with invasive SPAP, calculated during right heart catheterization, and a discordant PHT diagnosis (difference >10 mmHg between non-invasive and invasive SPAP) represent independent predictors of adverse outcomes (death, heart failure hospitalization, and reintervention) in patients with severe TR.

## 5. Preoperative Management of Tricuspid Regurgitation

Managing patients with tricuspid valve disease could be challenging due to their comorbidities, which can impact their clinical condition. Surgical risk may be affected by both central factors, such as right ventricular dysfunction, and peripheral factors, in particular hepatic and renal impairment. Current guidelines focus on the essential role of multidisciplinary discussions, starting from the preoperative risks of the patient, to carry out a tailored approach, including medical, surgical, and transcatheter strategies. Evaluating the risk of surgery for patients undergoing isolated tricuspid valve surgery may be challenging. In fact, the conventional score used for assessing “high-surgical risk patients”, like Euro SCORE II (European System for Cardiac Operative Risk Evaluation) or the STS score (Society of Thoracic Surgeons Risk Score), may be insufficient. In 2018, LaPar et al. [57] introduced the Clinical Risk Score (CRS) to predict perioperative mortality or adverse events after isolated tricuspid valve surgery. This score includes various preoperative characteristics of patients, such as age, gender, history of stroke, renal failure, lung disease, left ventricular function, New York Heart Association (NYHA) class, reoperation, type of surgery, and urgent or emergent status. Hence, the CRS might be useful to identify patients suitable for tricuspid valve surgery. Despite its potential, routine clinical use of the CRS remains uncommon. Wang et al. [59] analyzed and compared three commonly used risk calculators: (i) EuroSCORE II, (ii) the CRS, and (iii) the MELD score. The Model for End-Stage Liver Disease (MELD) score, frequently used for liver disease staging and follow-up, has been involved in the perioperative management of tricuspid valve disease due to hepato-renal dysfunction resulting from right heart failure. In his study, an author highlighted the key role of risk models in the decision-making process towards tricuspid valve surgery. Moreover, he showed the predominance of EuroSCORE II over the STS score for predicting operative mortality and post operative complications. The MELD score, instead, presents similar results to EuroSCORE II. Prof Dreyfus [60] and his staff developed a new risk score called the “TRI-SCORE”, including echocardiographic parameters, liver function, and clinical signs of right heart failure. In 2024, the TRI-SCORE underwent a significant upgrade compared to the 2022 version, dividing risk classes into low, intermediate, and high risk. A recent study by Adamo et al. examined patients undergoing tricuspid valve transcatheter procedures. They highlighted that patients with a TRI-SCORE ≥ 8 have a higher risk of post-procedural blood transfusions, acute kidney injury, new onset of atrial fibrillation, and in-hospital mortality and an increase in the length of post-procedural hospital stay. Hence, those patients should be carefully evaluated by the heart team to avoid performing futile procedures [61]. There are many differences between euroscore II and the TRI-SCORE when we consider the patient characteristics included in each respective score. Euroscore was developed to calculate the risk percentage for cardiac surgery patients, whereas the TRI-SCORE was specifically designed to calculate the risk percentage for patients with TR. The TRI-SCORE is more specific for TR patients because the Euroscore does not take into account the status of right heart function, the mechanism of TR, or the functional state of venous circulation, which includes the liver function index and the daily intake of diuretics. These factors contribute to the specificity of the TRI-SCORE. It should be noted that the two scores were developed for different purposes and at different times. In our opinion, after analyzing the differences, the TRI-SCORE is significantly more appropriate to use in patients with TR who are undergoing surgery. However, calculating both scores could provide a more comprehensive overall picture (Table 5 and Table 6).

## 6. Treatment Options

### 6.1. Open Approach

The management of surgical treatments for TR [62,63] includes assessing preoperative factors, like the patient’s comorbidities, and the anatomical characteristics of the tricuspid valve to determine the best surgical strategies between valve replacement and repair. Multimodal imaging is commonly used to choose the optimal treatment approach [64]. Nowadays, catheter-based technologies may have a pivotal role in device selection, guided by anatomical analysis. According to Carpentier’s classifications of mitral regurgitation, the assessment of the regurgitation mechanism, including annular, leaflet, and subannular components, should guarantee a standardized approach. For functional TR, annular dilation and leaflet tethering play a key role in surgical valve repair as a treatment option to restore valve competency. Valve replacement, instead, is preferred in advanced cases of ventricular dysfunction or geometric distortion, where repair may be ineffective. Surgery treatment remains the gold standard for low-risk patients [15,65]. Recently, studies suggested that isolated tricuspid surgery has a mortality rate <5% in low-risk patients, using a risk score model that includes parameters like age, heart failure severity, and renal function. In the case of leaflet tethering or ventricular dysfunction or remodeling, annuloplasty may be useful, or additional leaflet procedures may be needed for enhanced durability [66,67]. In these particular cases, a shift towards valve replacement occurred due to the high risk of re-intervention. The role of multimodal imaging for the choice of prosthesis used for tricuspid replacement is minimal, unlike repair procedures [68]. Surgery is recommended in the case of moderate or greater functional tricuspid valve regurgitation when left-sided valve surgery is needed, as current guidelines suggest. Also, surgery should be considered in the case of significant annular dilation (>40 mm or >24 mm/^m2^) regardless of the severity of TR in the case of left-sided surgery. The open approach can be performed via median sternotomy or a minimally invasive technique, either with the heart beating or arrested, depending on the surgeon’s preference and the patient’s comorbidities. Various surgical repair techniques have been explored over the years, though some have been abandoned due to surgical failures. (i) The Kay procedure, or bicuspidization technique, converts the trileaflet valve into a bicuspid one by folding the posterior leaflet and suturing the anteroposterior and posteroseptal commissure, resulting in a reduction in the orifice area. Nevertheless, this method does not eliminate the risk of damaging structures surrounding the tricuspid annulus, such as the coronary sinus. (ii) The Clover technique involves suturing the edge of the three leaflets together at their midpoints, creating a clover-shaped valve. This procedure is widely used for post traumatic lesions or myxomatous degeneration. (iii) De Vega annuloplasty is performed using a double- needle suture, starting from the posterior septal junction to the anterior septal junction, to narrow the annular orifice. Although it is a relatively straightforward procedure, long-term outcomes are limited because of the recurrence of moderate or greater regurgitation. iv) Ring annuloplasty should be considered a modified version of De Vega annuloplasty, where the ring guarantees a stabilization of the annulus, maintaining a more consistent shape and improving long-term outcomes by reducing the likelihood of reoperation. To achieve orifice area reduction, the sutures are placed from the anteroseptal commissure to the anterior and poster annulus, finishing at the center of the septal leaflet, avoiding the AV node and the conduction system. Carpentier was the first to introduce a rigid ring, but over time, semi-rigid and flexible rings have been utilized, yielding better hemodynamic results by accommodating the physiological movement of the tricuspid annulus. Studies have shown how the use of the ring provides significant advantages in decreasing TR degree [68,69].

When TV replacement is necessary, two different types of prosthesis may be implanted: biological and mechanical valves. Bioprosthetic valves do not require lifelong anticoagulation and have a low risk of thrombosis, and they allow the possibility of pacemaker implantation in the RV when needed. Although studies on the durability of biological valves are limited, bioprostheses in the TV position generally perform better than those in the mitral position, likely due to the lower pressure on the right side of the heart and the reduced mechanical shear stress on the cusps. Clinical data indicate that valve deterioration may occur after 7–10 years post implantation, with a younger patient age and a smaller valve size being key factors in early deterioration. On the other hand, mechanical valves, although rarely performed in the tricuspid position, are durable but have a higher incidence of intra-valvular thrombosis and thromboembolic events, such us pulmonary embolism, because of the lower pressure and blood velocity in the heart’s right chambers. Furthermore, once a mechanical prosthesis is implanted, right heart catheterization and PMK implantation become unfeasible. Additionally, the possibility of the transcatheter valve-in-valve procedure may be considered in the decision-making process regarding valve choice. Unlike aortic or mitral replacement, there is no specific age cut-off for the use of biological versus mechanical valves [70]. Early mortality outcomes after isolated tricuspid repair or replacement remain significant depending on the patient’s clinical condition, including factors such as age, NYHA class, atrial fibrillation, renal or hepatic failure, right heart dysfunction, pulmonary hypertension, and late presentation for surgery. Tricuspid valve replacement carries a greater risk of post operative pacemaker implantation compared to repair, primarily due to iatrogenic damage or compression of the septal leaflet, closed to AV node. A meta-analysis by Wang et al. reported a lower incidence of in-hospital complications associated with tricuspid repair, such as renal failure and pacemaker implantation. Conversely, there is an increased risk of stroke with unclear underlying mechanisms, highlighting the necessity for further studies. These findings have been supported by several studies, which demonstrate that long-term outcomes, including the need for reintervention, structural valve deterioration, valve thrombosis, and recurrent TV regurgitation, are significantly worse for valve replacement compared to repair. No substantial differences have been observed between biological and mechanical prostheses [71,72]. Finally, whenever feasible, tricuspid valve repair should be preferred over replacement with better early and late outcomes [73,74]. Predictive factors of 1-year mortality include age, chronic heart failure, cirrhosis, chronic kidney disease, malnutrition, emergency procedure, and preprocedural shock, as Kundi et al. [75] analyzed. The levels of these factors might be directly linked to greater adverse events, underlining how optimal surgical timing and patient selection play a key role in reducing peri-operative mortality rates [76]. Although isolated tricuspid surgery is linked to poorer early and late outcomes compared to left-sided interventions, especially in terms of mortality, untreated TV disease may lead to progressive right heart failure and repeated hospitalization. Promising data suggests how surgical treatments for isolated TV represent the optimal choice according to significant improvements in functional capacities of patients in long-term outcomes. A multidisciplinary heart team should be proposed to discuss the best approach based on the patient’s comorbidities, echocardiography, and right heart catheterization. Catheter-based technologies have recently been introduced as an alternative approach for patients at high and prohibitive risk. However, not all patients achieve optimal results, showing that further studies are needed and that patient selection represents a key element in treatment decision-making [77,78].

### 6.2. Percutaneous Approach

Percutaneous approaches include procedures on leaflets and the implantation of devices to target annular dilation and increase leaflet coaptation. Also, heterotopic and orthotopic valve replacement have been advocated in recent studies. The TEER procedure (i.e., TriClip) is the most common transcatheter edge-to-edge repair technique and it stands for the surgical Alfieri “clover technique”, a surgical annuloplasty option. Limited data are available for long-term outcomes, but TEER represents a valid alternative for high-risk patients as it is a safe and efficient treatment, at least in short-term follow-up. Procedural success is correlated with anatomical factors, such as a TV coaptation gap greater than 10 mm, leaflet tethering > 7 mm, a pacemaker-induced leaflet impingement, and a significant regurgitant orifice area, showing that valve morphology and the right ventricular ejection fraction should impact TEER outcomes. Nevertheless, right ventricular remodeling post-TEER has been observed, and its clinical implications remain uncertain [79]. The TRILUMINATE study (TRILUMINATE study with Abbott transcatheter clip repair system in patients with moderate or greater TR) highlighted how reaching a TR < 2+, assessed by echocardiography, leads to better outcomes at a 1-year follow-up in terms of mortality events and/or heart failure hospitalization. Some annuloplasty devices may be sufficient as standalone procedures in selected patients when the TR mechanism is associated with annular dilation without the involvement of leaflet motion or RV geometry. Multimodal imaging, including CT scan and TEE, are crucial for patient selection and procedural planning. Kodali et al. evaluated the 1-year outcomes of patients undergoing transcatheter valve repair using the PASCAL system (CLASPT II TR). Procedural success and the incidence of adverse events are directly correlated with the grade of residual TR. The PASCAL system revealed lower rates of complications and greater survival rates, including major cardiovascular events, functional status, and quality of life [80]. When anomalies of the RV geometry or TV components occur, it is mandatory to replace the valve, as pioneering studies regarding the use of percutaneous devices have shown. CT scan represents a starting point for patient selection, evaluating annular dimensions, RV anatomy, and vena cava positioning. Echocardiography and right heart catheterization are also helpful for excluding patients with a high risk of the procedure being unsuccessful due to advanced right heart failure or greater pulmonary resistance [81]. Therefore, for patients at high or prohibitive risk for surgery, or in the case of unsuitable anatomical structures, a transcatheter tricuspid valve replacement (TTVR) should be proposed for compassionate use. Recently, a percutaneous transfemoral TTVR (The EVOQUE System, TRISCEND II Trial) has been performed, showing favorable 1-year outcomes in terms of symptoms, right heart remodeling, mortality, and heart failure hospitalizations. Data show low mortality and rehospitalization rates, a relevant absence of residual TR, and better clinical conditions of patients, according to the NYHA classification. These data suggest how the EVOQUE system could become a promising alternative treatment for fragile patients [82]. The same results have been illustrated by Blasco-Turrion et al. analyzing patients undergoing heterotopic transcatheter treatments. This study involved high-risk patients unsuitable for surgery, who were treated with the “Tricvalve system”, a caval valve implantation. The 1-year follow-up showed better clinical improvement in terms of quality of life, NYHA functional class, and a lower mortality rate [76]. Although the inclusion criteria of the three trials mentioned above were different, as shown in Table S1, the exclusion characteristics were similar (see Table S2). In particular, conditions of scarce compliance, with advanced left and right ventricular failure or poor clinical conditions, represent exclusion criteria from both surgical and transcatheter procedures. Focusing on the trials’ results, it became clear that, regardless of the procedure analyzed, early treatment might lead to the short and long-term outcomes being improved, and all strategies should be implemented before severe systemic or cardiac complications arise (Tables S1 and S2, Supplementary Materials).

## 7. Discussion

The 2021 ESC guidelines emphasize the importance of symptoms and right ventricular enlargement as key factors in determining the need for treatment. Surgical intervention is advised for patients with “severe isolated tricuspid regurgitation who experience significant symptoms or show progressive right ventricular dilation or dysfunction”. Similarly, the American Heart Association (AHA) and American College of Cardiology (ACC) guidelines align closely with the recommendations outlined in the European guidelines. Preoperative screening is also emphasized as a critical step to improve patient outcomes. Additionally, the European guidelines have recently included transcatheter treatment (TTVI) as an option for patients at high surgical risk or those deemed unsuitable for surgery. For patients with advanced cardiomyopathy or significant dysfunction of the left or right ventricle, or in cases of pulmonary hypertension (sPAP > 60 mmHg), medical management remains the primary approach [83]. Numerous studies have demonstrated a significant association between the effective regurgitant orifice area (EROA) and adverse outcomes across various clinical contexts [84,85]. According to the American Society of Echocardiography (ASE) and the European Association of Cardiovascular Imaging (EACVI), severe tricuspid regurgitation (TR) is characterized by an EROA ≥ 0.40 cm^2^ and a regurgitant volume (RVol) ≥ 45 mL. However, patients undergoing transcatheter tricuspid valve interventions (TTVIs) frequently present anatomical regurgitant areas substantially exceeding the threshold EROA of 0.40 cm^2^. Consequently, an expanded classification system that includes “massive” and “torrential” TR—both linked to adverse clinical outcomes—has been proposed [59,60]. In cases of severe TR, a thorough evaluation of the right ventricle (RV) should be performed while the patient is in a euvolemic state. This evaluation should encompass standard echocardiographic measurements of RV size and function, alongside assessments of RV morphology, functional capacity, and tissue remodeling [61]. RV strain analysis via 2D echocardiography or cardiac magnetic resonance imaging (CMR) is less load-dependent than tricuspid annular plane systolic excursion (TAPSE) and provides greater sensitivity in detecting early RV dysfunction and predicting clinical outcomes. Although the RV ejection fraction can be measured using modalities such as CMR, 3D echocardiography, or cardiac computed tomography (CCT), it may overestimate RV systolic function in cases of severe TR as it does not account for the interplay between RV contractility and afterload [62]. To address this limitation, the TAPSE/systolic pulmonary artery pressure (SPAP) ratio has emerged as a valuable non-invasive marker of RV–pulmonary artery coupling. A TAPSE/SPAP ratio < 0.31 mm/mmHg is indicative of poor prognosis in severe TR and other conditions [63,64]. Notably, a recent propensity-matched analysis indicated that patients with moderate RV dysfunction (TAPSE 13–17 mm) may experience improved outcomes following TTVI [65,66,67,68,69,70,71,72,73,75,76,77,78,79,80,81,83,84,85,86,87,88,89]. The presence of contractile reserve, assessed through pharmacological or physical stress, holds prognostic significance in patients with pulmonary hypertension (PHT) and severe baseline RV dysfunction. Further research is warranted to clarify the role of stress imaging in the management of severe TR. Right heart catheterization remains the gold standard for evaluating the severity and etiology of PHT, assessing parameters such as pulmonary vascular resistance, the right atrial pressure/pulmonary capillary wedge pressure ratio, the pulmonary artery pulsatility index, and PHT reversibility [66,67,68,69,70,71,72,73,74,75,76,77,78,79,80,81,83,84,85,86,87,88,89]. Moreover, TAPSE adjusted for afterload using invasive SPAP measurements obtained during catheterization, as well as discordant PHT diagnoses (a > 10 mmHg difference between non-invasive and invasive SPAP), have been identified as independent predictors of adverse outcomes, including mortality, heart failure hospitalization, and reintervention, in patients with severe TR. Isolated tricuspid valve surgery is infrequently performed because of the high mortality rate compared to other cardiac operations. Hence, surgical treatment for patients, before meeting class I indications for isolated TR, may be considered in an early phase to improve outcomes. Early surgery is correlated with a reduced rate of morbidity and a low rate of intraoperative mortality in contrast to a higher rate of mortality rate associated with class I indications. Isolated TV surgery has traditionally yielded suboptimal outcomes, with operative mortality rates hovering around 10%, even in contemporary studies. Nevertheless, current guidelines approached isolated TV surgery in a conservative manner. Analyzing data derived from TR surgery associated with left-sided valve disease might provide an interesting starting point for further studies on the timing of isolated TR surgery. As suggested by Cao et al., in cases of left-sided valve surgery with concomitant mild or moderate TR disease, opting not to treat TR may lead to cardiac-related mortality in the long term and a progression to more than severe TR. Moreover, early mortality events were similar in both the repair and unrepair groups, and there was even a higher cardiopulmonary bypass time in the treated group [90]. As previously mentioned, the onset of severe tricuspid regurgitation after left heart surgery can negatively impact the patient’s prognosis and quality of life. When severe tricuspid valve disease occurs, a reintervention may be necessary, with very high operative risks. In these cases, the use of minimally invasive techniques should be considered to reduce perioperative risks, guaranteeing a lower mortality rate and successful results following TV repair [91,92]. Minimally invasive approaches offer several advantages compared to open surgery, including a lower risk of renal complications, reduced device implantation, and lower mortality rates. They also result in better outcomes for patients, such as less pain, fewer wound infections, and earlier mobilization [93]. Right minithoracotomy allows for excellent exposure to the right atrium and tricuspide valve, enabling surgery on a beating heart. Two alternatives for tricuspid valve surgery are the arrested heart (AH) technique under cardiopulmonary bypass and beating heart (BH) surgery. Currently, there is no consensus on which technique is superior. However, a study by Russo et al. [94] indicated that patients undergoing beating heart surgery had better long-term survival rates and a lower 30-day mortality rate (6.2% in the AH group versus 5% in the BH group). A study proposed by Wang et al. [70] focused on the characteristics and outcomes of surgery in patients who underwent TV surgery in an early stage compared with class I indications. Their data showed an operative mortality rate of 0% in early surgery versus 8% in class I indications, suggesting that surgery should be performed as soon as possible [95,96]. It might be necessary to obtain a good selection of patients, to achieve a good hemo-dynamic state in the preoperatory phase, and to carry out correct management in the post operatory period. Moreover, the composite morbidity rate, including acute kidney injury and prolonged ventilation, presents a significant difference between the two groups, with 8% in the early surgery group versus 41% in class I, underlying that comorbidities play a key role in surgical outcomes. Waiting for class I indications allow for the development of several comorbidities, leading to a higher surgical risk and a more aggressive approach. Despite this, isolated TV surgery remains infrequent, showing a discrepancy between the procedure’s potential benefits and its utilization. In the cardiac surgery setting, the idea of early surgery has already been applied to other valvular diseases, highlighting how patients undergoing early surgery present better outcomes [97,98,99]. For example, some trials showed that early surgery improves outcomes in asymptomatic patients with severe aortic stenosis. Similarly, several studies suggested that left ventricular dilation from volume overload due to aortic and mitral regurgitation is linked with worse outcomes and may require immediate surgery [100,101]. Overall, when is the correct time for surgery? Certainly, factors such as an older age, a higher prevalence of symptoms, prior cardiac surgeries and devices, heart failure, atrial fibrillation, chronic lung disease, renal impairment, and left and right heart systolic dysfunction may contribute to increasing both the surgical and long-term risks, and an early surgery should be performed before these comorbidities occur. However, in patients with isolated severe TR under surveillance, it should be unpredictable when symptoms arise, supporting the idea of earlier surgery to enhance outcomes [102,103]. Modifiable risk factors should be treated with medical therapies to reduce preoperatory risk. Additionally, in the surgery setting, valve repair is recommended when feasible over replacement to improve outcomes because of the limited durability of prostheses and the risk of infection. The management of patients affected by isolated severe TR includes the administration of medical therapies [82,104]. Drugs such as diuretics, rhythm control, antithrombotic, and pulmonary hypertension medical therapies might play a role in reducing secondary TR severity and ventricular remodeling and function. Recently, transcatheter management of TV developed as an alternative strategy for patients at high or prohibitive risk for surgery. The results from an international registry showed positive outcomes, with a 30-day mortality rate of 3,6% and an acceptable 1-year survival rate [105]. However, in an experienced center, surgical treatment of TV should be preferred according to the surgical morbidity and mortality rates. Nevertheless, further investigations are needed to fully explore the efficacy and safety of transcatheter tricuspid interventions and to define the criteria for its applicati [106].

## 8. Gaps in Evidence

**Figure 4:**
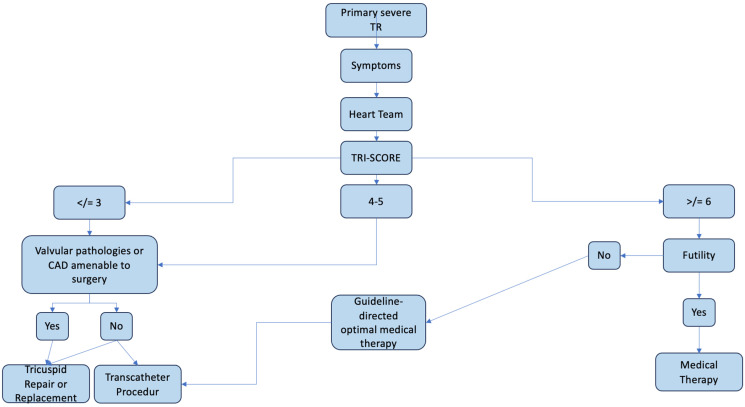
Hypothetic flowchart of decisional process for treatment of TR.

Tricuspid regurgitation (TR) has been an underestimated topic for such a long time, largely due to the relative rarity of patients affected by this condition. Treatment of tricuspid valve disease is often performed with other valvular defects or other cardiac pathologies. The scarce number of isolated TR cases has resulted in limited studies and meta-analyses in this field, causing a significant gap in the literature. In this paper, we reported the most recent data on this topic. As previously mentioned, the TRI-SCORE is currently the most effective tool for guiding TR treatment decisions. Moreover, according to this score, patients undergoing TR surgery before the onset of clinical and cardiac failure present significantly lower post operative mortality rates. Focusing on the three trials, TRILUMINATE, CLASP II, and TRISCEND II, we found that, despite their differences in enrollment criteria, the exclusion criteria share common characteristics. The results of these trials highlight promising outcomes, with a notable reduction in mortality rates among patients with intermediate TRI-SCOREs (4–5) undergoing tricuspid interventions. All evidence suggests that TR should be treated at an early stage rather than waiting for clinical and echocardiographic deterioration. Patients with a TRI-SCORE higher than 6, as previously discussed, are at a high peri-operative risk with a greater likelihood of surgical complications. However, due to the lack of specific studies, it is evident that future research should fill this literature gap, especially regarding the optimal time of intervention for both surgical and transcatheter approaches. Another important aspect to discuss is the *futility* of the intervention itself. When does the treatment of tricuspid valve disease become futile? Over time, the literature has transitioned from focusing on a compassionate intervention to a routine procedure, but the guidelines have not yet reached a common consensus. Furthermore, it might be possible that establishing the correct time of TV intervention should eliminate the concept of futility. Analyzing the trials mentioned above, it can be seen that the exclusion criteria include patients with poor compliance, for example, those with a high TRI-SCORE (>6 or >8). For these patients, even the transcatheter approach becomes futile due to scarce outcomes; thus, medical therapy is recommended as the primary option. In conclusion, future studies should be focused on tricuspid valve disease and the optimal time of intervention to enhance the decision-making process and to identify the best technique based on patient characteristics. This approach may provide clinical benefits for patients, avoiding discussions of futility in the near future (Figure 4).

## 9. Conclusions

In conclusion, isolated tricuspid surgery remains an infrequent procedure with a higher risk of morbidity and mortality compared to coronary or other valve procedures. Early surgical intervention was associated with lower mortality during follow-up in contrast to patients with a late referral, which remains the class I indication according to the guidelines. These data suggest that an earlier approach to treat severe isolated TR is preferred rather than a watchful waiting approach until patients meet the class I indication. Considering the systemic impairment generally associated with TR, especially in the later stages of presentation, patients with isolated tricuspid valve disease remain extremely challenging for heart teams to treat as the available strategies should be tailored to the patient to improve outcomes. Ideally, centers able to provide both transcatheter and surgical approaches should be favored considering the need for a dedicated “tricuspid team”.

## Figures and Tables

**Table 1:** Echocardiographic measures of RV. DTI: Doppler tissue imaging; EDV: end-diastolic volume; ESV: end-systolic volume; FAC: fractional area change; LS: longitudinal strain; PLAX: parasternal long-axis view; PW: pulsed wave; RIMP: right ventricular index of myocardial performance; RV: right ventricular; RVEF: right ventricular ejection fraction; RVOT: right ventricular outflow tract; SD: standard deviation; TAPSE: tricuspid annular plane systolic excursion.

RV Size Parameter	Mean +/− SD	Normal Range
RV basal diameter, mm	33 +/− 4	25–41
RV mid diameter, mm	27 +/− 4	19–35
RV longitudinal diameter, mm	71 +/− 6	59–83
RVOT PLAX diameter, mm	25 +/−2.5	20–30
RVOT proximal diameter, mm	28 +/− 3.5	21–35
RVOT distal diameter, mm	22 +/− 2.5	17–27
RV EDV, mL/mÇ		
Men	61 +/− 13	35–87
Women	53 +/− 10.5	32–74
RV ESV, mL/mÇ		
Men	27 +/− 8.5	10–44
Women	22 +/− 7	8–36
**RV Function Parameter**	**Normal Range**	**Abnormal**
TAPSE, mm	24 +/− 3.5	<17
DTI S’, cm/s	14.1 +/− 2.3	<9.5
Free wall LS, %	−29 +/− 4.5	>-20
RIMP (PW Doppler)	0.25 +/− 0.085	>0.43
RIMP (DTI)	0.38 +/− 0.08	>0.54
FAC, %	49 +/− 7	<35
RVEF, %	58 +/− 6.5	<45

**Table 2:** classification of tricuspid regurgitation.

Classification	Etiologies
**Primary TR**	
**Degenerative Disease**	Prolapse Flail
**Congenital**	Ebstein’s Anomaly Leaflet clefts
**Acquired**	Rheumatic disease (usually with left-side disease) Infective endocarditis Endomyocardial fibrosis Carcinoid disease, serotonin active drugs Traumatic (blunt chest injury, laceration) Iatrogenic
**Functional TR**	
**Ventricular secondary** **TR**	Left heart diseases (left ventricular dysfunction or left heart valve diseases) resulting in pulmonary hypertension Primary pulmonary hypertension Secondary pulmonary hypertension (e.g., chronic lung disease, pulmonary thromboembolism, left-to-right shunt) Right ventricular dysfunction from any cause (e.g., myocardial diseases, ischemic heart disease, chronic right ventricular pacing)
**Atrial secondary** **TR**	Atrial fibrillation Heart Failure with preserved ejection fraction
**Cardiac tumors**	Right atrial myxomas
**Cardiac implantable electronic device (CIED) induced TR (~5% of patients)**	
**Primary CIEDinduced** **TR**	CIED caused by direct interaction of the lead on the valve leaflets)
**Secondary CIEDinduced** **TR**	Incidental CIED, with TR due to functional etiologies or pacing related remodeling

**Table 3:** classification of tricuspid regurgitation; + mild, ++ moderate, +++ severe, − not present.

Parameter	Atrial FTR	Ventricular FTR	CIED Related	Primary TR Prolapse (I) RHD (IIIA)
**Leaflet tethering**	−	+++	++	− −
**Leaflet restriction**	−	Systole	Systole/Diastole	− Diastole
**RA/TA dilatation**	+++	++	+/−	++ ++
**RV dilatation**	+/−	+++	+/−	+/− +/−
**RV dysfunction**	+/−	+++	+/−	+/− +/−

**Table 4:** Grades of tricuspid regurgitation and quantitative parameters.

Parameters	Mild	Moderate	Significant/ Moderate-Severe	Severe	Massive	Torrential
**Vena contracta**	<3 mm	3–6.9 mm	6–6.9 mm	7–13 mm	14–20 mm	≥21 mm
**EROA**	20 mm^2^	20–29 mm^2^	30–39 mm^2^	40–59 mm^2^	60–79 mm^2^	≥80 mm^2^
**Regurgitant volume**	<15 mL	15–29 mL	30–44 mL	45–59 mL	60–74 mL	≥75 mL

**Table 5:** Differences between EuroScore II and TRI-SCORE.

Score	EuroScore II	Tri-Score
Patients anamnesi	Patient-related factors Age and sex Height and weight Pulmonary disease Diabetes status Extracardiac arteriopathy Neurological or musculoskeletal dysfunction On dialysis Last serum creatinine Brain-natriuretic peptide Serum albumin Cardiac-related factors Symptomatic status NYHA CCS LV function Recency and size of last myocardial infarct Systolic PA pressure Active endocarditis Previous cardiac surgery Operation-related factors Urgency Elective Urgent Emergency Salvage Type of procedure(s) performed in detail Times of Bypass Cross-clamp Deep hypothermic arrest Selective cerebral perfusion	Age ≥ 70 years NYHA functional Class III–IV Right-sided heart failure signs Daily dose of furosemide ≥ 125 mg Glomerular filtration rate < 30 mL/min Elevated total bilirubin Left ventricular ejection fraction Moderate/severe right ventricular dysfunction Mechanism of tricuspid regurgitation

**Table 6:** New TRI-Score. ^1^ TTVR: transcatheter valve repair. ^2^ After adjustment for sex, age, atrial fibrillation, and comorbidities. ^3^ AHR: adjusted hazard ratio, presented with 95% confidence interval and *p* value resulting from comparison with conservative treatment.

	In-Hospital Mortality		2-Year Survival Rate			2-Year Survival Rate, Pooled Comparison		AHR ^3^	
	SURGERY 9.6%	TTVR ^1^ 2.5%	CONSERVATIVE 1217 Patients 327 Events 71%	SURGERY 551 Patients 111 Events 77%	TTVR 645 Patients 118 Events 69%	UNADJUSTED	ADJUSTED ^2^	SURGERY	TTVR
**LOW TRI-SCORE (≤3)** ***N* = 764**	2.7%	0.7%	433 patients 79%	183 patients 93%	148 patients 87%	*p* < 0.001	*p* = 0.006	0.35 [0.18–0.69] *p* = 0.002	0.65 [0.32–1.31] *p* = 0.23
**INTERMEDIATE** **TRI-SCORE** **(4–5)** ***N* = 800**	185 9.2%	256 2%	359 patients 71%	185 patients 80%	256 patients 71%	*p* = 0.13	*p* = 0.15	0.73 [0.47–1.15] *p* = 0.14	0.69 [0.44–1.09] *p* = 0.11
**HIGH TRI-SCORE** **(≥6)** ***N* = 849**	183 16.9%	241 4.3%	425 patients 61%	183 patients 58%	241 patients 56%	*p* = 0.66	*p* = 0.48	1.21 [0.87–1.70] *p* = 0.26	0.98 [0.68–1.43] *p* = 0.90

## Supplementary Materials

The following supporting information can be downloaded at https://www.mdpi.com/article/10.3390/jcm14145063/s1, Table S1: Inclusion criteria; Table S2: Exclusion criteria.

## Abbreviations

2D	two-dimensional
3D	three-dimensional
AF	atrial fibrillation
CCT	cardiac computed tomography
CIED	cardiac implantable electronic device
CMR	cardiac magnetic resonance
EROA	effective regurgitant orifice area
HF	heart failure
NOAC	non-vitamin K antagonist oral anticoagulants
PHT	pulmonary hypertension
RA	right atrium
RV	right ventricle
SPAP	systolic pulmonary artery pressure
STR	secondary tricuspid regurgitation
TAPSE	tricuspid annular plane systolic excursion
TEE	transesophageal echocardiography
TR	tricuspid regurgitation
TTE	transthoracic echocardiography
T-TEER	tricuspid transcatheter edge-to-edge repair
TTVI	transcatheter tricuspid valve intervention
TTVR	transcatheter tricuspid valve replacement
TV	tricuspid valve
