# Supplementary material for: Isolated Tricuspid Regurgitation: When Is Surgery Appropriate? A State-of-the-Art Narrative Review
Source: J Clin Med. 2025 Jul 17;14(14):5063. doi: 10.3390/jcm14145063 (PMC12295814; doi:10.3390/jcm14145063)
Supplement: Supplementary file 1 [file jcm-14-05063-s001.zip › jcm-3656860-supplementary.pdf]

Trial	TRILUMINATE (TRICLIP)	CLASP II TR (PASCAL)	TRISCENDT II (EVOQUE)
**Inclusion criteria**	1. Adequate Treatment and Stability:<br>The site's local heart team must confirm that the subject has received appropriate treatment according to applicable standards and has been stable for at least 30 days. This includes:<br>- Optimized medical therapy for tricuspid regurgitation (TR), such as diuretics.<br>- Medical and/or device therapy for conditions like mitral regurgitation, atrial fibrillation, coronary artery disease, and heart failure.<br>The Executive Committee (EC) will verify that the subject has been adequately treated medically.<br><br>2. Symptomatic Severe TR Despite Optimal Treatment:<br>- The subject must exhibit symptoms of severe TR despite optimal treatment as outlined above.<br>- TR severity must be determined through a qualifying transthoracic echocardiogram (TTE) and confirmed by the Echocardiography Core Lab (ECL).<br>- The ECL may also request a transesophageal echocardiogram to confirm the etiology of TR.<br>- Note: If any cardiac procedure(s) are performed after eligibility determination, TR severity must be reassessed 30 days after the procedure(s).<br><br>3. Surgical Risk:<br>- The cardiac surgeon from the site's local heart team must concur that the patient has an intermediate or higher estimated risk of mortality or morbidity with tricuspid valve surgery.<br><br>4. Functional Status:<br>- The subject must have a New York Heart Association (NYHA) Functional Class of II, III, or ambulatory Class IV.<br><br>5. Access Feasibility:<br>- The TriClip™ implanting Investigator must determine that femoral vein access is feasible and can accommodate a 25 French catheter.<br><br>6.Age Requirement:<br>- The subject must be 18 years of age or older at the time of consent.<br><br>7. Informed Consent:<br>- The subject must provide written informed consent before undergoing any trial-related procedure.	1. Age Requirement: The patient must be 18 years of age or older.<br>2. Persistent Symptoms Despite Medical Therapy: Based on the assessment of the local Heart Team, the patient exhibits signs or symptoms of tricuspid regurgitation (TR) or has a history of heart failure hospitalization due to TR, despite receiving medical therapy.<br>3. Severity of TR: The patient has severe or greater tricuspid regurgitation.<br>4. Functional Status or Recent Hospitalization: The patient is classified as New York Heart Association (NYHA) Class II-IVa or has been hospitalized for heart failure within the past 12 months.<br>5. Surgical Risk: The patient is considered to have an intermediate or higher estimated risk of mortality with tricuspid valve surgery, as determined by the cardiac surgeon in agreement with the local Heart Team.<br>6. Informed Consent and Compliance: The patient is capable of providing informed consent, adhering to protocol procedures, and attending follow-up visits as required.	1. Severity of TR: The patient must have at least severe functional and/or degenerative tricuspid regurgitation (TR).<br>2. Symptoms or History of TR: The patient shows signs or symptoms of TR despite optimal medical therapy (including stable oral diuretics, unless there is a documented history of intolerance) or has a history of heart failure hospitalization related to TR.<br>3. Anatomical Suitability: The patient's anatomy must be deemed appropriate for the Evoque valve.

Tab S1. Inclusion criteria

Trial	TRILUMINATE (TRICLIP)	CLASP II TR (PASCAL)	TRISCENDT II (EVOQUE)
**Exlusion criteria**	1. Pulmonary Hypertension: Systolic pulmonary artery pressure (sPAP) > 70 mmHg or fixed pre-capillary pulmonary hypertension, as confirmed by right heart catheterization (RHC).<br>2. Uncontrolled Hypertension: Severe uncontrolled hypertension defined as Systolic Blood Pressure (SBP) ≥ 180 mmHg and/or Diastolic Blood Pressure (DBP) ≥ 110 mmHg.<br>3. Prior Tricuspid Valve Procedures: Any previous tricuspid valve procedure that could interfere with the placement of the TriClip™ device.<br>4. Recent Valve Interventions: Indication for left-sided (e.g., severe aortic stenosis, severe mitral regurgitation) or pulmonary valve correction within the prior 60 days.<br>- Note: Patients with concomitant mitral and tricuspid valve disease may undergo mitral regurgitation treatment and be reassessed for the trial after 60 days.<br>5. Pacemaker/ICD Leads: Leads that prevent the appropriate placement of the TriClip™ device.<br>6. Tricuspid Valve Stenosis: Defined as a tricuspid valve orifice of ≤ 1.0 cm^2 and/or a mean gradient ≥ 5 mmHg, as measured by the Echocardiography Core Lab (ECL).<br>7. Severely Reduced LVEF: Left ventricular ejection fraction (LVEF) ≤ 20%.<br>8. Tricuspid Valve Anatomy: Leaflet anatomy unsuitable for clip implantation or effective TR reduction due to factors such as:<br>- Calcification in the grasping area.<br>- A severe coaptation defect (> 2 cm).<br>- Severe leaflet defects that hinder device placement.<br>- Ebstein's anomaly, characterized by a normal annulus position with valve leaflets attached to the right ventricle's walls and septum.<br>9. Imaging Limitations: Tricuspid valve anatomy not evaluable by transthoracic (TTE) and transesophageal echocardiography (TEE).<br>10. Active Infections or Degenerative Leaflets: Active endocarditis, active rheumatic heart disease, or leaflet degeneration due to rheumatic disease (e.g., noncompliant or perforated leaflets).<br>11. Recent Cardiac Events:<br>- Myocardial infarction (MI) or unstable angina within the prior 30 days. - Percutaneous coronary intervention (PCI) within the prior 30 days.<br>12. Hemodynamic Instability: Defined as systolic pressure < 90 mmHg (with or without afterload reduction), cardiogenic shock, or the need for inotropic support, intra-aortic balloon pump, or other hemodynamic support devices.<br>13. Cerebrovascular Events: Cerebrovascular accident (CVA) within the prior 90 days.<br>14. Chronic Dialysis: Requirement for chronic renal replacement therapy.<br>15. Bleeding or Clotting Disorders: Presence of bleeding disorders, hypercoagulable states, active peptic ulcers, or active gastrointestinal (GI) bleeding.<br>16. Antiplatelet/Anticoagulant Therapy: Contraindication, allergy, or hypersensitivity to both dual antiplatelet and anticoagulant therapy.<br>- Note: Contraindication to one therapy (either antiplatelet or anticoagulant, but not both) is not an exclusion criterion.<br>17. Active Infection: Current infection requiring antibiotic therapy. Patients may enroll 30 days after completing antibiotics, provided there is no active infection.<br>18. Allergy to Device Materials: Known allergy or hypersensitivity to materials used in the device.<br>19. Intracardiac or Venous Abnormalities: Presence of intracardiac, inferior vena cava (IVC), or femoral venous thrombus, mass, or vegetation.<br>20. Limited Life Expectancy: Estimated life expectancy of less than 12 months.<br>21. Concurrent Clinical Trials:<br>- Participation in another clinical trial that has not completed its primary endpoint.<br>- Participation in another clinical investigation related to valvular heart diseases.<br>22. Pregnancy or Nursing:<br>- Pregnant or nursing individuals, or those planning pregnancy during the clinical investigation follow-up period.<br>- Women of childbearing potential must have a negative pregnancy test within 7 days of the baseline visit and use effective contraception (e.g., intrauterine devices, hormonal methods, or double-barrier methods).<br>23. Other Conditions: Any anatomic, comorbid, medical, social, or psychological condition that, in the investigator's opinion, could:<br>- Limit the subject's ability to participate in the study.<br>- Affect compliance with follow-up requirements.<br>- Compromise the scientific validity of the study results.	1. Tricuspid Valve Evaluation: Anatomy of the tricuspid valve not assessable by transthoracic (TTE) or transesophageal echocardiography (TEE).<br>2. Anatomical Barriers: Tricuspid valve anatomy that prevents proper deployment and function of the device.<br>3. Advanced Heart Failure: Patient with refractory heart failure requiring advanced intervention, such as a left ventricular assist device (LVAD) or heart transplantation (ACC/AHA Stage D heart failure).<br>4. Pacemaker/Defibrillator Leads: Presence of trans-tricuspid pacemaker or defibrillator leads that:<br>- Interfere with proper tricuspid regurgitation (TR) reduction due to lead-leaflet interaction.<br>- Were implanted in the right ventricle (RV) within the last 90 days prior to enrollment.<br>5. Primary Non-Degenerative Tricuspid Disease: Diagnosis of primary non-degenerative tricuspid valve disease.<br>6. Previous Tricuspid Surgery: Prior repair or replacement of the tricuspid valve that could interfere with the placement of the PASCAL device.<br>7. Significant Coronary Disease: Clinically significant, untreated coronary artery disease that requires revascularization, unstable angina, acute coronary syndrome, or a recent myocardial infarction.<br>8. Intracardiac Abnormalities: Significant mass, thrombus, or vegetation within the heart as assessed by the core lab.<br>9. Venous Thromboembolism: Deep vein thrombosis (DVT) or pulmonary embolism (PE) within the last 180 days.<br>10. Recent Stroke: History of stroke within the recent past.<br>11. Active GI Bleeding: Ongoing gastrointestinal bleeding.<br>12. Infiltrative Cardiomyopathy/Valvulopathy: Conditions such as carcinoid, amyloidosis, sarcoidosis, hemochromatosis, or significant congenital heart diseases, including atrial septal defect, RV dysplasia, and arrhythmogenic RV.<br>13. Emergent or Planned Surgery: Need for urgent surgery for any reason, planned cardiac surgery within the next 12 months, or planned percutaneous cardiac procedures within the next 90 days.<br>14. Recent Cardiovascular Procedures:<br>- Percutaneous coronary, intracardiac, or endovascular intervention within the last 30 days prior to enrollment.<br>- Carotid surgery within the last 30 days prior to enrollment.<br>- Direct current cardioversion within the last 30 days prior to enrollment.<br>- Leadless RV pacemaker implantation within the last 30 days prior to enrollment.<br>- Cardiac surgery within the last 90 days prior to enrollment.<br>15. Valve Disease: Severe aortic, mitral, and/or pulmonic valve stenosis or regurgitation.<br>16. Carotid Artery Disease: Known history of untreated severe symptomatic carotid stenosis or asymptomatic carotid stenosis.<br>17. Endocarditis or Recent Infection: Active endocarditis or recent infection that required antibiotic therapy.<br>18. Chronic Obstructive Pulmonary Disease (COPD): Requirement for continuous home oxygen therapy.<br>19. Pregnancy: Current pregnancy or plans to become pregnant within the next 12 months.<br>20. Life Expectancy: Concurrent medical conditions with an estimated life expectancy of less than 12 months, as determined by the Investigator.<br>21. Participation in Other Studies: Active participation in another investigational biologic, drug, or device clinical study.<br>22. Consent and Follow-Up: Presence of medical, social, or psychological conditions that prevent appropriate consent or follow-up, or if the patient is under guardianship.<br>23. Vulnerability: Any patient deemed to be vulnerable.	1. Left Ventricular Function: Left ventricular ejection fraction (LVEF) less than 25%.<br>2. Right Ventricular Dysfunction: Severe right ventricular (RV) dysfunction.<br>3. Pacemaker Dependency: Dependence on a pacemaker without any viable alternative pacing options.<br>4. Prior Tricuspid Procedures: History of tricuspid surgery or intervention.<br>5. Heart Transplantation: Previous heart transplantation.<br>6. Renal Function: Estimated glomerular filtration rate (eGFR) of ≤25 mL/min/1.73 m^2 or the need for chronic renal replacement therapy.<br>7. Anatomical Considerations: Anatomical constraints that prevent proper device delivery, deployment, or function.

Tab S2. exclusion criteria
